# Machine Learning Analysis of Image Data Based on Detailed MR Image Reports for Nasopharyngeal Carcinoma Prognosis

**DOI:** 10.1155/2020/8068913

**Published:** 2020-02-21

**Authors:** Chunyan Cui, Shunxin Wang, Jian Zhou, Annan Dong, Fei Xie, Haojiang Li, Lizhi Liu

**Affiliations:** ^1^Sun Yat-sen University Cancer Center, State Key Laboratory of Oncology in South China, Collaborative Innovation Center for Cancer Medicine Guangdong Key Laboratory of Nasopharyngeal Carcinoma Diagnosis and Therapy, Guangzhou 510060, China; ^2^Zhongshan School of Medical, Sun Yat-sen University, Guangzhou, Guangdong 510080, China

## Abstract

We aimed to assess the use of automatic machine learning (AutoML) algorithm based on magnetic resonance (MR) image data to assign prediction scores to patients with nasopharyngeal carcinoma (NPC). We also aimed to develop a 4-group classification system for NPC, superior to the current clinical staging system. Between January 2010 and January 2013, 792 patients with recent diagnosis of NPC, who had MR image data, were enrolled in the study. The AutoML algorithm was used and all statistical analyses were based on the 10-fold test. Primary endpoints included the probabilities of overall survival (OS), distant metastasis-free survival (DMFS), and local-region relapse-free survival (LRFS), and their sum was recorded as the final voting score, representative of progression-free survival (PFS) for each patient. The area under the receiver operating characteristic (ROC) curve generated from the MR image data-based model compared with the tumor, node, and metastasis (TNM) system-based model was 0.796 (*P*=0.008) for OS, 0.752 (*P*=0.053) for DMFS, and 0.721 (*P*=0.025) for LRFS. The Kaplan-Meier (KM) test values for II/I, III/II, IV/III groups in our new machine learning-based scoring system were 0.011, 0.010, and <0.001, respectively, whereas those for II/I, III/II, IV/III groups in the TNM/American Joint Committee on Cancer (AJCC) system were 0.118, 0.121, and <0.001, respectively. Significant differences were observed in the new machine learning-based scoring system analysis of each curve (*P* < 0.05), whereas the *P* values of curves obtained from the TNM/AJCC system, between II/I and III/II, were 0.118 and 0.121, respectively, without a significant difference. In conclusion, the AutoML algorithm demonstrated better prognostic performance than the TNM/AJCC system for NPC. The algorithm showed a good potential for clinical application and may aid in improving counseling and facilitate the personalized management of patients with NPC. The clinical application of our new scoring and staging system may significantly improve precision medicine.

## 1. Introduction

Nasopharyngeal carcinoma (NPC), which is a malignant cancer arising in the epithelium of the nasopharynx, is a prevalent form of cancer in a number of populations, including those in South China, Southeast Asia, the Arctic, the Middle East, and North Africa [1–3]. It was estimated that 60,600 new cases of NPC were diagnosed in mainland China in 2015, accounting for 40% of NPC cases worldwide [4]. Radiotherapy is the primary treatment for NPC. In recent years, intensity-modulated radiotherapy (IMRT) has been extensively used by virtue of its lower normal tissue doses and more uniform target doses when compared to conformal radiotherapy [5–8]. This has led to improved disease outcomes due to a higher local tumor control rate. In fact, distant metastasis is now the predominant reason for treatment failure in patients with NPC [9]. Advances in diagnostic and therapeutic techniques have improved the management and treatment of NPC [10–12]. Due to its high spatial resolution for examination of soft tissues, magnetic resonance imaging (MRI) has been extensively used as the optimal imaging modality for the assessment of local, regional, and intracranial infiltration of NPC in clinical practice [13]. The usefulness of MRI for stage assignment and disease prognosis has also been reported [12].

The TNM staging system of the American Joint Committee on Cancer (AJCC) [14] is generally accepted as the most widely used tool for cancer staging and plays a key role in guiding treatment and determining the prognosis of NPC in clinical practice. According to recent research, the current 8^th^ edition of the AJCC staging system for NPC is still not completely satisfactory, though it enables a more accurate prediction of treatment outcomes than the 7^th^ edition [15]. The demarcation line between the early and late stages (stages II and III) is still not well defined [16]. This leads to confusion in the choice of treatment by physicians. Moreover, patients in the same stage may require different treatments based on their prognoses. Since the TNM/AJCC system is based on factors manually selected from conventional radiographic data, a vast amount of anatomic structural data may be neglected. Because the significance of such data is unknown, neglecting these data may be a limitation of the current TNM system. Therefore, detailed MR image reports containing larger amounts of potentially significant features for prognosis as well as new statistical tools with the ability to analyze large amounts of data with good predictive performance are both urgently needed.

Machine learning (ML) is the best solution to the problem discussed above. ML is able to create reasonable generalizations, classify previously unseen data, discover patterns, or predicts new directions based on observed data using multifarious artificial intelligence and statistical models [17]. Many ML methods have been reported in studies of conditions such as lung cancer, breast cancer, and Alzheimer's disease. However, the state-of-the-art ML technique is “automatic machine learning” (AutoML), which has the ability to automatically select the ML classifier with the best performance to suit the data. There are several excellent open-source AutoML algorithms, including XGBoost, Deep learning (DL), and Light GBM. AutoML also includes common models such as Lasso and Ridge Regression, Random Forest, and Naivebayes. In summary, AutoML is easy to program and its use is feasible in clinical applications.

Based on the above premise, we utilized image data from detailed MR image reports and used ML to make prognosis predictions. We also compared the prognostic value of the predictions made using ML to that of the traditional TNM/AJCC system. To the best of our knowledge, almost no medical professionals have performed similar research. Therefore, our study would be the first step in the exploration of this issue.

## 2. Materials and Methods

### 2.1. Patient

The Sun Yat-sen University Cancer Center Institutional Review Board approved this retrospective study and waived the requirement for informed consent from the patients. We enrolled 3,814 patients with newly diagnosed NPC in the Sun Yat-sen University Cancer Center between January 2010 and January 2013. Of these patients, 2,973 were excluded due to incomplete medical records. Of the remaining 841 patients, 24 with distant metastasis and 5 without neck MRI and 20 cases combined with other tumors were excluded. Finally, 792 patients were included in our study.

The eligibility criteria were as follows: (1) pathologically diagnosed NPC; (2) no evidence of distant metastasis; (3) receipt of standard IMRT treatment, the details of which were determined by radiation therapists, physicians, and highly qualified physicists (platinum-based chemotherapy was used as routine treatment for NPC); (4) no primary tumor in other parts of the body; and (5) complete imaging and clinical data. The exclusion criteria were as follows: (1) presence of primary tumors in other parts of the body and (2) failing to complete radiotherapy for physical reasons during the course of treatment. All patients completed a pretreatment examination including a complete medical history, physical examination, hematology and biochemistry profiles, chest X-ray, abdominal computed tomography, and MRI of the neck and nasopharynx.

### 2.2. MR Imaging and Image Analysis

MRI studies were performed using a 1.5 T (Signa CV/*i* General Electric Healthcare, Chalfont St. Giles, United Kingdom) or 3.0 T system (Siemens Magnetom Tim Trio, Erlangen, Germany) with standard scan procedures. Scanning range included the saddle pool to the lower edge of the sternal collarbone with combined head/neck coils. Non-contrast-enhanced axial, coronal, and sagittal plane T1-weighted images (WI) and axial T2-WI were procured. The following scanning parameters were used: T1WI in the axial, coronal, and sagittal planes (fast-spin-echo (FSE), TR/TE = 500–600/10–20 ms, field of view (FOV) = 22 cm, frequency matrix = 256 × 512). T2-WI in the axial plane (FSE, TR = 4000–6000 ms, TE = 95–110 ms, FOV = 22 cm, frequency matrix = 256 × 512). The contrast agent gadolinium diethylenetriaminepentaacetic acid was injected at a dose of 0.1 mmol/kg. T1-weighted axial and sagittal sequences and fat-suppressed coronal imaging were performed sequentially using the same parameters before contrast injection. The scanning section thickness was 5 mm, and the section gaps were 1 mm.

Two radiologists specializing in head-and-neck cancers for over 10 years separately evaluated all MRI materials and gave detailed MR image reports with the average time of 15–20 minutes, and the stage for each NPC case was determined according to the 8^th^ edition of the AJCC staging system [14]. A review of invasion of other anatomical structures based on lymph node information was also performed. Disagreements were resolved by consensus.

### 2.3. Follow-Up

After treatment, patients were followed up every three months for the first two years and every six months thereafter for 5 years. The terminal outcomes were distant metastasis-free survival (DMFS), local-regional relapse-free survival (LRFS), progression-free survival (PFS), and 5-year overall survival (OS).

### 2.4. Machine Learning

**Step 1 .**

Tenfold cross-validation (10 CV): Patients' data were randomly divided 10-fold without duplication to simulate the 10 times of external validations.

**Step 2 .**

Feature reduction is crucial for reducing time consumption by different classifiers and for more clinical feasibility. First, using the RMRe feature of the R Package with default setting, the top 20 most important features were chosen and ranked separately for OS/DMFS/LRFS. Second, in addition to the generalized linear model (GLM) (including Lasso and Ridge) algorithm with grid hyperparameter search in R's H2O package, which has an extremely fast run (<120 s), we progressively added features 1 to 20 separately; the most important features corresponded with the biggest average area under curve (AUC) of 10 CVs (attached code sample: gusting_proper_number_of_variables.r). The detailed description is shown in Supplementary [Supplementary-material supplementary-material-1].

**Step 3 .**

Using the AutoML algorithm of the H2O package with the above important variable, the total learning time was set to 7200 s to ensure that sufficient models were trained under different algorithms. The algorithm and model with the highest average AUC of 10 CVs were eventually chosen. Similarly, the TNM system, as the control group, used only two features with the T- and N-stage (attached code sample: automl.r). The demo R code is demonstrated in Supplementary [Supplementary-material supplementary-material-1] (http://docs.h2o.ai/h2o/latest-stable/h2o-docs/automl.html for AutoML).

**Figure 1:**
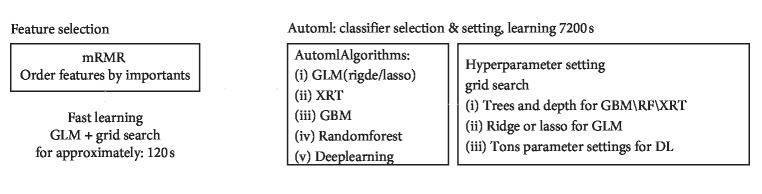
Flow chart of feature selection used in this study. First step: feature selection to reduce features. Second step: AutoML is run. AutoML performs hyperparameter search (parameters such as tree and depth in the flow chart are representative examples) over a variety of H2O algorithms to deliver the best model. The hyperparameters of AutoML supported by grid search are listed in Supplementary [Supplementary-material supplementary-material-1]. Abbreviations: mRMR, minimum redundancy maximum correlation; GLM, generalized linear model; XRT, extreme random tree; GBM, gradient boosting machine; RF, random forest; DL, deep learning.

Figure 1 depicts the flow chart of our feature selection. The detailed hyperparameter setting and grid search are shown in Supplementary [Supplementary-material supplementary-material-1].

### 2.5. Statistical Analysis

We compared data between the image data group and the TNM system-control group. Prognostic statistics in the above 10 CV experiments including 10 AUCs, test error, and specificity, along with their average and standard deviations (SDs) for predicting OS, DMFS, and LRFS, were calculated separately for the image data and TNM system-control groups. Paired *t*-test was used to compare the two groups because they shared the same group information declared in the 10 CV section. *P* < 0.05 was considered statistically significant [18].

Probabilities with a value of 1 to 100 distribution generated by the final model were considered for scoring OS, DMFS, and LRFS separately. The sum of the three scores (PFS = OS + DMFS + LRFS) was considered the standard voting point of PFS, because in clinical practice, PFS events are defined as any event pertaining to OS, DMFS, and LRFS. Then this voting point for PFS was further cut off using the receiver operating characteristic (ROC) curve method to generate four new staging curves. To create four survival curves, we required three cutoffs, and the most appropriate method for obtaining the cutoff value is ROC curve analysis. The Youden-index was calculated using the ROC curve. For the first time, a total of 792 cases from the entire data were used to calculate the first ROC cutoff value (0.986), and those with a score higher than this were classified as stage IV; others with a cutoff value lower than this value were used to calculate the second ROC cutoff value (0.643) to determine stage III cases. Similarly, the third ROC cutoff value (0.270) was used to define stage I/II. The Kaplan-Meier survival analysis was used to compare the OS, DMFS, LRFS, and PFS staging curves between our new machine learning-based scoring system and the 8^th^ TNM staging system.

Statistical analyses were performed using R 3.1.2 (http://www.R-project.org), with the main R package including mRMRe, H_2_O, survival, Hmisc, and stats.

## 3. Results

### 3.1. Patient Characteristics

Of the 792 patients, 576 were male and 216 were female (male/female ratio, 2.7 : 1). The median age was 45 years (range, 11–78).

Table 1 lists the patient characteristics. The median follow-up period was 55.6 months (range, 1.2–83.4 months). Overall, 94 patients (11.9%) developed distant metastases, 78 patients (9.8%) relapsed, 87 patients (11.0%) died, and 25 patients (3.2%) had both distant metastases and relapses. The 5-year survival rates for the entire cohort were as follows: OS, 89.0%; DMFS, 88.1%; and LRFS, 90.2%.

### 3.2. Prognostic Performance

**Figure 2:**
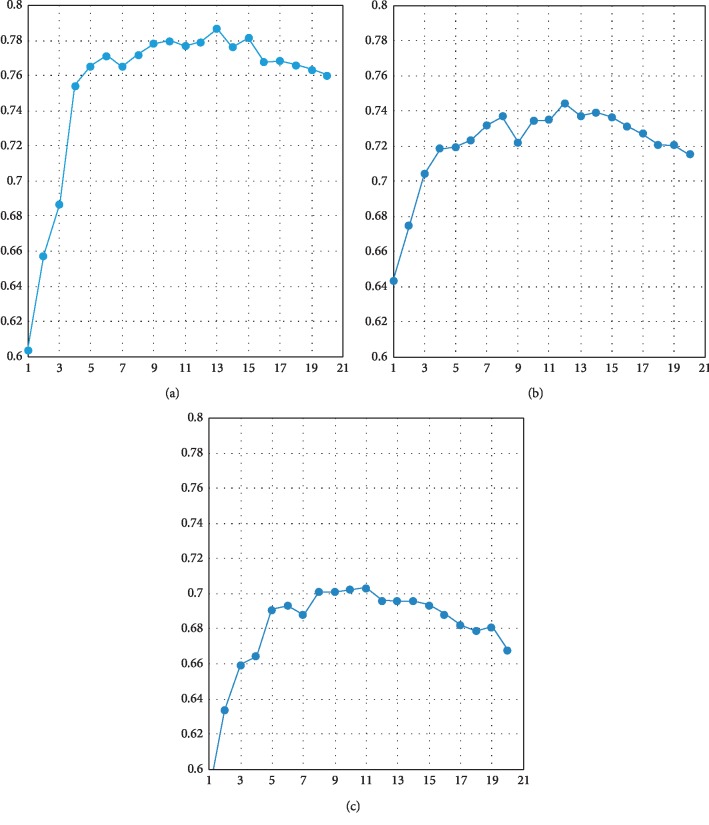
Feature selection as performed by AutoML. (a) The important imaging findings of OS with 13 variables, ranked according to their importance, are listed; the best AUC is selected. (b) The important imaging findings of DMFS with 12 variables, ranked, are listed; the best AUC is selected. (c) The important imaging findings of LRFS with 11 variables, ranked, are listed; the best AUC is selected.

The feature selection was performed by AutoML, shown in Figure 2. All in all, important imaging findings including OS with 13 variables, DMFS with 12 variables, and LRFS with 11 variables are listed in Table 2 and ranked according to their importance. During AutoML learning, each algorithm corresponds to multiple models since grid search automatically generates hyperparameter, and the best AUC is finally selected. The final algorithm of AutoML included OS, ridge regression with parameter (lambda = 0.004, nlambda = 30); DMFS, GBM (Trees = 49, min depth = 3, max depth = 7, min leaves = 4, max leaves = 18), and LRFS, GLM ridge (lambda = 0.002, nlambda = 30).

The mean AUC of image data- and TNM system-based ML model is illustrated in Table 3. The average AUC of 10 CVs for OS, DMFS, and LRFS results obtained from the image data-based model was 0.796, 0.752, and 0.721, respectively, which was higher than 0.712, 0.693, and 0.617, respectively, obtained from the TNM system-based model. The *P* values were 0.008, 0.053, and 0.025 for OS, DMFS, and LRFS, respectively. The average test errors of 10 CVs for OS, DMFS, and LRFS results from image data-based model were lower than those obtained from the TNM system-based model, and the *P* values were 0.006, 0.011, and 0.006, respectively. The average specificity of 10 CVs for OS, DMFS, and LRFS results from the image data-based model was greater than those from the TNM system-based model, and the *P* values were 0.006, 0.011, and 0.006, respectively.

**Figure 3:**
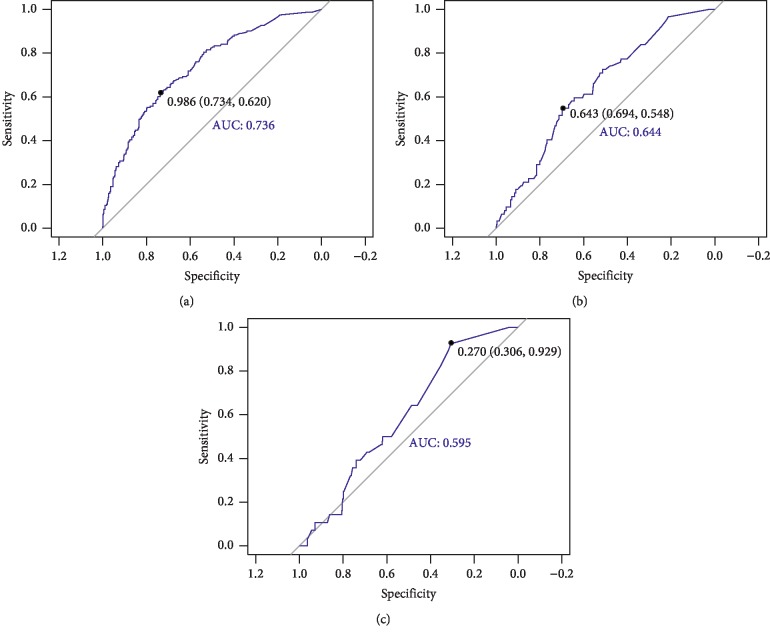
Receiver operating characteristics of Cox regression. (a) We used PFS fraction and PFS to establish the ROC curve, and the best cutoff value was 0.986 (highest Youden-index), which is regarded as the first cutoff value. We considered the first cutoff value over 0.986 for the new staging IV. Next, we used the remaining patients to draw the ROC curve and calculate the remaining two cutoff values. (b) The second cutoff value is not less than 0.643, which is in accordance with staging III. (c) The third cutoff value is not less than 0.270, which is in accordance with staging I and II. Stage IV PFS%≥0.986, Stage III PFS%≥0.643, Stage II PFS%≥0.270, and Stage I PFS% <0.270.

Each patient received three voting points representing OS, DMFS, and LRFS. The sum of these voting points represented the PFS voting points and its ROC curve (Figure 3). Using the above PFS voting point system, we created a ROC curve and established three cutoff points, and the best cutoff value obtained was 0.986 (highest Youden-index). The second cutoff value was greater than 0.643, which is in accordance with staging III. The third cutoff value was greater than 0.270, in accordance with staging I and II.

**Figure 4:**
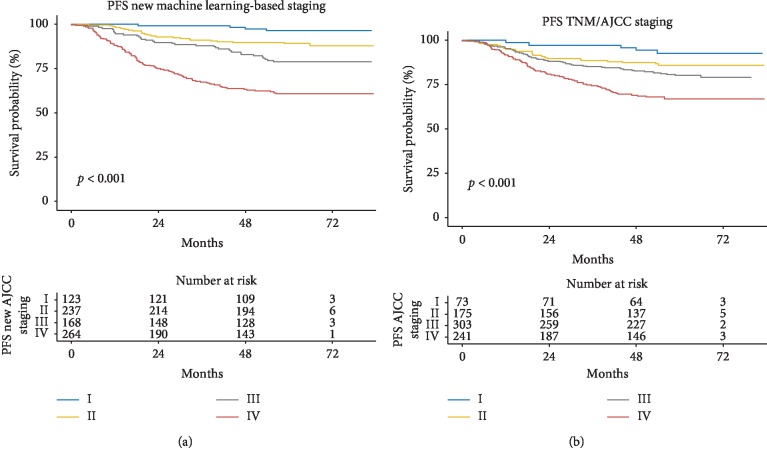
Analysis of Kaplan-Meier survival curve for the estimation of progression-free survival (PFS): (a) new scoring system (b) AJCC staging.

Further, the survival curves estimated by Kaplan-Meier survival analysis of PFS are shown in Figure 4, and the main statistical data are represented in Table 4. Significant differences were observed in the new machine learning-based scoring system for each ROC curve (*P* < 0.05). On the contrary, in the TNM/AJCC system, the *P* value of curves between II/I and III/II was 0.118 and 0.121, respectively, which was not significantly different. Moreover, the patient number distributed as per the new scoring system was more balanced compared to the TNM/AJCC system.

## 4. Discussion

The image data-based model had higher AUC and specificity and lower test error than the TNM system-based model for prediction of OS, DMFS, and LRFS. We thus conclude that image data from detailed MR image reports outperform the TNM system in prognosis prediction for NPC. More interestingly, we used the OS, DMFS, and LRFS prediction probabilities to assign a PFS probability. Using this measure, we established a new scoring staging system, which was proven to be superior to the current TNM/AJCC system.

The TNM/AJCC system is an important tool for the prediction of prognosis and for guiding treatment strategies for patients in different risk groups. It is used to evaluate the disease based on the range of local invasion, regional lymphatic spread, and distant metastasis, which are regarded the major prognostic factors for NPC. Fundamentally, the TNM/AJCC system is based on several important anatomic structures that are the most significant and well-accepted features of the disease on MR images. The system thus considers multiple types of prognosis (OS, DMFS, LRFS, and PFS). Nevertheless, it is difficult to meet all independent statistical conditions for the same factor at the same time without neglecting others. According to recent studies, the current 8^th^ edition of the TNM/AJCC is still not completely satisfactory in risk segregation and survival prediction [16, 19, 20]. Recently, an increasing number of studies have confirmed the inadequacy of the current TNM/AJCC system using “nomograms.” Such studies highlight the urgent need for a better prognostic determination system for risk categorization of individual patients [21, 22].

We created a scoring system for NPC based on detailed MR image reports using ML. We used the scoring system to predict the probabilities of OS, DMFS, and LRFS for each patient and then added the above three probabilities to calculate a PFS probability, which was used to create a new scoring staging system. We divided the patients into four groups using our new scoring system and found that our new machine learning-based scoring system was better than the original AJCC system. Although our study was the first to design such a scoring system, our findings confirm its feasibility. Our scoring system even outperforms the current AJCC system in certain respects. For instance, the new scoring staging system tends to assign patients in the same stage with worse prognoses to the advanced stage. This improves the accuracy of the individualized treatment. Furthermore, the new scoring system may be used to assign prognoses for patients in the same AJCC stage. This would enable physicians to prescribe more precise individualized treatments for patients in the same stage. Our scoring system suggested that physicians should use higher-dose therapy for patients in the same stage who had worse prognoses. In contrast, the scoring system suggested that patients with better prognoses should be treated based on the old guideline.

The new machine learning-based scoring system reported here was based on detailed MR image reports from a world-class nasopharyngeal cancer research center. As mentioned earlier, we adopted a completely unique report analysis method that has never been reported previously. The image data derived from the detailed MR image reports provide more precise and abundant information than the current 8^th^ edition stage NPC. These data contribute to the prognostic system, but only under the condition that the influential factors can be recognized and selected from such a large amount of data.

Current statistical methods used for staging rely mainly on Cox regression. To optimize its predictive performance, several studies have added more factors to the TNM system to build a scoring system using a nomogram based on Cox regression. However, Cox regression cannot be used to effectively select important features in a stepwise manner when applied to a large number of variables and large amounts of data.

ML, which is a booming field at the intersection of computer science, statistics, data mining, and optimization [23], appears to be the preferred option for the resolution of the challenges described above. Due to its strong foundation in statistical theory, ML has become increasingly popular and acceptable when compared to conventional clinical algorithms [24, 25]. ML is mathematically based on multifold systems, such as the 10-fold system, as well as cross-validation to simulate different sets of data. AutoML, which is the ML method we chose for feature selection, has been demonstrated to be robust and to outperform traditional approaches in a previous report [26]. The use of AutoML has become increasingly prevalent in business, science, and other disciplines. Unlike other ML methods, AutoML can be used to automatically suit the data and select the machine learning classifier with the best performance. Interestingly, AutoML can be used to find the optimal combination of prediction algorithms using a process called Stacked Ensembles. Due to the lower time demanded, higher prognostic performance, and easy application without the need for expert programming knowledge, AutoML has the potential to be used by individuals who are not ML experts. It was thus easy to establish the new scoring system without the use of complicated steps. Our approach was shown to be accurate and stable in a clinical application and has the potential for use in precision medicine.

Our new scoring system had advantages when compared to the existing scoring systems based on nomogram. For example, the nomogram scoring is fixed when the variables are determined, while the new scoring system can be used to assign separate prognostic scores for OS, DMFS, and LRFS and to calculate a comprehensive prognostic score. Therefore, it is convenient for doctors to assess and stage patients based on the different prognostic indicators in the new system in the clinic. For patients with the same PFS probability, doctors can shorten the review time of patients with high LRFS probability and enhance the treatment of patients with high DMFS probability by comparing their OS, DMFS, and LRFS probability.

This study has some limitations. First, the new method ignores the time to event as compared with the existing survival analysis. Second, we do not have a testing set currently due to small data volume and unbalanced data; a large external data volume can be used as an external testing set in future studies. Third, our new scoring system was established using only image data from detailed MR image reports. Considering that a number of other prognostic predictors of NPC have been reported, including the presence of Epstein-Barr virus DNA and the C-reactive protein/albumin ratio (CRP/Alb), the prognostic performance of our scoring system can be improved substantially by including the above-mentioned variables.

## 5. Conclusion

Image data from detailed MR image reports enabled better prognostic performance in predicting treatment outcomes for NPC than that achieved using the TNM/AJCC system. We created a completely new scoring system, which is a step toward the enhancement of image data-based clinical predictions and precision oncology.

## Figures and Tables

**Table 1:** Patient characteristics.

Characteristics	*n* (%)	OS (%)	DMFS (%)	LRFS (%)
Total	792 (100%)	89.0	88.1	90.2
Gender				
Male	576 (72.7%)	91.7	91.7	93.1
Female	216 (27.3%)	97.3	96.5	97.1
Age				
≥45	397 (50.1%)	92.3	92.8	94.4
<45	395 (49.9%)	96.7	95.3	95.7
Histology				
WHO I	5 (0.6%)	99.9	99.9	99.9
WHO II	41 (5.2%)	99.1	99.1	99.4
WHO III	746 (94.2%)	90.0	89.1	90.9
T stage (a)				
T1	204 (25.8%)	99.0	98.5	98.2
T2	97 (12.2%)	98.6	98.6	99.1
T3	296 (37.4%)	96.6	95.7	96.5
T4	195 (24.6%)	94.8	95.3	96.3
N-stage (a)				
N0	182 (23.0%)	98.7	98.6	98.9
N1	438 (55.3%)	94.4	94.3	93.8
N2	113 (14.3%)	97.7	96.8	98.4
N3	59 (7.4%)	98.1	98.4	99.1
Clinical stage (a)				
1	73 (9.2%)	99.9	99.7	99.6
2	175 (22.1%)	98.9	98.5	98.2
3	303 (38.3%)	96.6	95.6	96.6
4	241 (20.4%)	93.7	94.3	95.7

Abbreviations: WHO, World Health Organization; (a) according to the 8th AJCC/UICC.

**Table 2:** All important imaging higher ranked imaging findings.

ID	Variables	Numbers	5 years surv	*P*	Important
OS					
1	Jugular foramen	770/22	89.32/53.13	<0.001	1.000
2	Paranasal sinuses	695/97	90.63/71.70	<0.001	0.966
3	Central necrosis of retropharyngeal LNs	696/96	89.58/78.75	0.002	0.893
4	Steep hill	494/298	92.08/81.96	<0.001	0.891
5	Meninges	764/28	89.57/48.05	<0.001	0.791
6	Invasion of carotid sheath	655/137	90.16/79.47	0.001	0.666
7	Extracapsular invasion of cervical LNs	735/57	89.09/77.70	0.002	0.630
8	Number of lower cervical LNs	0∼11	—	<0.001	0.488
9	Bilateral of parapharyngeal space	691/101	89.96/76.36	<0.001	0.336
10	Number of retropharyngeal LNs	0∼6	—	0.002	0.332
11	Pressure of carotid sheath	775/17	88.43/80.00	0.075	0.327
12	Number of upper cervical LNs	0∼23	—	<0.001	0.256
13	Posterior nasal meatus invasion	648/144	89.96/80.61	<0.001	0.205
DMFS					
1	Number of cervical LNs	0∼34	—	<0.001	1.000
2	Meninges	764/28	88.81/51.63	<0.001	0.462
3	Posterior nasal meatus invasion	648/144	90.01/77.16	<0.001	0.396
4	Bilateral of retropharyngeal LNs	575/217	91.14/78.30	<0.001	0.387
5	Infratemporal fossa	771/21	88.61/52.84	<0.001	0.386
6	Extracapsular invasion of cervical LNs	735/57	88.45/78.19	0.007	0.250
7	Central necrosis of retropharyngeal LNs	696/96	88.97/78.49	0.002	0.242
8	Medial cartilage of ear	315/477	90.73/85.69	0.034	0.234
9	Musculus longus colli	513/279	91.24/81.06	<0.001	0.231
10	Jugular foramen	770/22	88.43/62.20	<0.001	0.204
11	Sphenoid sinus	700/92	89.51/74.02	<0.001	0.197
12	Number of upper cervical LNs	0∼23	—	<0.001	0.182
LRFS					
1	Clustering of LNs	602/190	91.18/83.08	0.003	1.000
2	Pharyngeal recess	61/731	97.83/88.65	0.058	0.817
3	Paranasal sinuses	695/97	91.37/74.03	<0.001	0.765
4	Central necrosis of retropharyngeal LNs	600/192	91.02/83.98	0.004	0.540
5	Jugular foramen	770/22	90.03/64.62	<0.001	0.531
6	Bilateral of cervical LNs	317/475	93.85/86.11	0.001	0.366
7	Bilateral of retropharyngeal LNs	575/217	91.89/82.07	<0.001	0.309
8	Tensor velum palatine muscle	366/426	92.82/86.27	0.005	0.293
9	Bone of ear	755/37	89.83/79.78	0.038	0.193
10	Pressure of carotid sheath	775/17	89.70/72.51	0.033	0.145
11	Infratemporal fossa	771/21	89.78/72.96	0.012	0.137

*P*: log-rank; LNs = lymph nodes; number (none/yes or invasion); HR (—) for continue variables; important (standardize): while 1.000 is the most important. Variables are selected by mRMR.

**Table 3:** The mean AUC of image data-based and TNM system-based ML model.

	OS	DMFS	LRFS
Image data (AUC)	0.796 (0.044)	0.752 (0.042)	0.721 (0.052)
TNM system (AUC)	0.712 (0.064)	0.693 (0.050)	0.617 (0.073)
*P* value (AUC)	0.008	0.053	0.025
Image data (test error)	0.208 (0.037)	0.271 (0.052)	0.287 (0.050)
TNM system (test error)	0.326 (0.052)	0.346 (0.031)	0.413 (0.047)
*P* value (test error)	0.006	0.011	0.006
Image data (specificity)	0.721 (0.061)	0.576 (0.114)	0.540 (0.153)
TNM system (specificity)	0.405 (0.132)	0.383 (0.060)	0.174 (0.010)
*P* value (specificity)	0.006	0.011	0.006

The average performance of the two models is reported with standard deviation in the parenthesis.

**Table 4:** Kaplan-Meier (KM) test on II/I, III/II, and IV/III in ML-groups and TNM AJCC groups.

PFS AJCC staging	Number	Events	Events/number (%)	5 years surv	*P* value^*∗*^	
AutoML-based system						
I	123	4	3.25	0.964	—	—
II	237	26	10.97	0.891	0.011	II/I
III	168	34	20.24	0.788	0.010	III/II
IV	264	99	37.50	0.608	<0.001	IV/III
TNM-based system						
I	73	5	6.85	0.927	—	—
II	175	24	13.71	0.860	0.118	II/I
III	303	58	19.14	0.803	0.121	III/II
IV	241	76	31.54	0.670	<0.001	IV/III

^*∗*^ *P* values as KM way to compare cure II/I, III/II, IV/III.

## Data Availability

The data of the MRI and other measurements are stored in our RDD. These data used to support the findings of this study are available from the corresponding author upon request.

## Supplementary Materials

Supplementary Materials Supplementary material 1: the detailed hyperparameter setting and grid search are shown. Supplementary material 2: the demo R code is demonstrated.
