# Supplementary material for: Machine Learning Analysis of Image Data Based on Detailed MR Image Reports for Nasopharyngeal Carcinoma Prognosis
Source: Biomed Res Int. 2020 Feb 21;2020:8068913. doi: 10.1155/2020/8068913 (PMC7054759; doi:10.1155/2020/8068913)
Supplement: Supplementary Materials — Supplementary material 1: the detailed hyperparameter setting and grid search are shown. Supplementary material 2: the demo R code is demonstrated. [file 8068913.f1.zip › 8068913.f1/Supplementary material 1.docx]

From program page:

<http://docs.h2o.ai/h2o/latest-stable/h2o-docs/automl.html>

and

http://docs.h2o.ai/h2o/latest-stable/h2o-docs/grid-search.html

**Random Grid Search Parameters**

AutoML performs hyperparameter search over a variety of H2O algorithms in order to deliver the best model. In AutoML, the following hyperparameters are supported by grid search.

There are many parameters here, but all of them could be access the detail settings and range from their document page.

Take a parameter “stopping_metric” for example: from the following page we could access the full setting about it: [http://docs.h2o.ai/h2o/latest-stable/h2o-docs/search.html?q=stopping_metric]

### Common Hyperparameters

- fold_assignment
- fold_column
- max_runtime_secs
- offset_column
- stopping_metric
- stopping_rounds
- stopping_tolerance
- weights_column

### Deep Learning Hyperparameters

- activation
- adaptive_rate
- average_activation
- balance_classes
- categorical_encoding
- classification_stop
- class_sampling_factors
- col_major
- distribution
- elastic_averaging_moving_rate
- elastic_averaging_regularization
- elastic_averaging
- epochs
- epsilon
- fast_mode
- force_load_balance
- hidden_dropout_ratios
- hidden
- initial_biases
- initial_weights
- initial_weight_distribution
- initial_weight_scale
- input_dropout_ratio
- l1
- l2
- loss
- max_after_balance_size
- max_categorical_features
- max_w2
- missing_values_handling
- momentum_ramp
- momentum_stable
- momentum_start
- nesterov_accelerated_gradient
- overwrite_with_best_model
- quantile_alpha
- quiet_mode
- rate_annealing
- rate_decay
- rate
- regression_stop
- replicate_training_data
- reproducible
- rho
- score_duty_cycle
- score_interval
- score_training_samples
- score_validation_samples
- score_validation_sampling
- seed
- shuffle_training_data
- single_node_mode
- sparse
- sparsity_beta
- standardize
- target_ratio_comm_to_comp
- train_samples_per_iteration
- tweedie_power
- use_all_factor_levels
- variable_importances

### DRF Hyperparameters

- categorical_encoding
- mtries

### GBM Hyperparameters

- categorical_encoding
- col_sample_rate
- distribution
- huber_alpha
- learn_rate_annealing
- learn_rate
- max_abs_leafnode_pred
- pred_noise_bandwidth
- quantile_alpha
- rand_family
- rand_link
- startval
- tweedie_power

### GLM Hyperparameters

- alpha
- lambda
- missing_values_handling
- seed
- standardize
- theta
- tweedie_link_power
- tweedie_variance_power

### Naïve Bayes Hyperparameters

- compute_metrics
- eps_prob
- eps_sdev
- laplace
- min_prob
- min_sdev
- seed

### Shared Tree Hyperparameters

**Note**: The Shared Tree hyperparameters apply to DRF and GBM.

- balance_classes
- class_sampling_factors
- col_sample_rate_change_per_level
- col_sample_rate_per_tree
- histogram_type
- max_after_balance_size
- max_depth
- min_rows
- min_split_improvement
- nbins_cats
- nbins_top_level
- nbins
- ntrees
- sample_rate_per_class
- sample_rate
- seed
