# Supplementary material for: Machine Learning Analysis of Image Data Based on Detailed MR Image Reports for Nasopharyngeal Carcinoma Prognosis
Source: Biomed Res Int. 2020 Feb 21;2020:8068913. doi: 10.1155/2020/8068913 (PMC7054759; doi:10.1155/2020/8068913)
Supplement: Supplementary Materials — Supplementary material 1: the detailed hyperparameter setting and grid search are shown. Supplementary material 2: the demo R code is demonstrated. [file 8068913.f1.zip › 8068913.f1/Supplementary material 2. demo_code_automl.pdf]

```
#automl parameter
#train for 10 folds CV , running two hour for all OS/DMFS/LRFS
#then get a list order by mean AUC of 10 folds CV
#find the best one


#Automl running
m4 <- h2o.automl(x = c(1:tempx), y = y, training_frame =
deepTrain2,leaderboard_frame=deepTrain2,validation_frame =deepTrain2,

                          max_runtime_secs=7200,
                          ,fold_column=setFoldName        #10 folds CV
                          ,stopping_metric ="AUC"
                          ,stopping_tolerance=0.005
                          ,stopping_rounds =3


);


#get a list of mean AUC of 10 folds CV    of all models
automlMaxAuc=0
automlAuc=c()
for(i in 1:nrow(testobj@leaderboard)){

  modelname=testobj@leaderboard[i,1]

  if(length( unlist(strsplit(x=modelname,split="Ensemble")) )==1 ){

    thismodel=h2o.getModel(modelname)

    tempaaaa=as.numeric(thismodel@model$cross_validation_metrics_summary[2,1])
    automlAuc=rbind(automlAuc,c(modelname,tempaaaa))
    if(tempaaaa>automlMaxAuc){
      h2omethod=thismodel
      automlMaxAuc=tempaaaa
    }

  }

}#for

#list of mean AUC of 10 folds CV
automlAuc=as.data.frame(automlAuc)
```

#bestAUC:automlMaxAuc
